# Impact of COVID-19 on pets and pet owners: A survey conducted in selected veterinary clinics in Accra, Ghana

**DOI:** 10.1016/j.heliyon.2024.e37328

**Published:** 2024-09-04

**Authors:** Godwin Dogbey, Amos Dugah, Richard Kwamena Abbiw, Anthony Agbolosu, Kweku Asare-Dompreh, Theophilus Odoom, Allen Okine, Jonathan Amakye-Anim, Hope Richard Otsyina, Ben Enyetornye

**Affiliations:** a School of Veterinary Science, University for Development Studies, Nyankpala, Ghana; b West African Centre for Cell and Molecular Biology of Infectious Pathogens, University of Ghana, Legon, Ghana; c School of Veterinary Medicine, College of Basic and Applied Sciences, University of Ghana, Legon, Ghana; d Accra Laboratory, Veterinary Services Directorate, Ghana; e La Veterinary Hospital, Labadi, Accra, Ghana

**Keywords:** Covid-19, Ghana, Pets, Pet owners

## Abstract

The study explores the impact of Covid-19 on pets and their owners using a structured questionnaire. One hundred fifty-seven (157) respondents were recruited from six veterinary clinics at strategic points within Accra. Only a third of owners gave their pet(s) special attention out of fear of contracting Covid-19 from their pet(s). 68.2 % of the respondents were interested in learning healthy pet-keeping tips from the electronic media and evident in their willingness (75.2 %) to embrace tele-veterinary services; a potential drift from traditional in-person way of providing veterinary services in Ghana. The decrease in household income (46.5 %), loss of employment for at least one person in the household (17.2 %) and associated spike in prices of pet related commodities affected the feeding of pets in about 44 % of respondents and ability to afford veterinary pet products (17.2 %). This is reflected in a 12.7 % reduction in the ability of pet owners to afford veterinary services. The observations made could be used as a basis for future research exploring the pet culture in Ghana and its evolution as a direct consequence of the pandemic.

## 1 Introduction

Covid-19 was declared a pandemic on March 11, 2020, by the World Health Organization (WHO) [1]. Countries adopted partial and complete lockdown measures to curtail the rapid spread of Covid-19 disease [2,3]. The pandemic and its associated actions negatively affected several sectors of global and national economies [4] living a long-lasting aftermath. Amid all these economic upheavals, households and their pets were not exempted from the repercussions of the lockdown restrictions of Covid-19. Like humans, the associated Covid-19 protective measures presented a radical shift in animal behaviour and normal patterns. Pets were met with the challenge of having to be walked less often and socialized minimally with others, raising numerous welfare concerns. Pet abandonment due to the fear that pets might play a role in the spread of Covid-19 was of great concern. Moreover, accessibility to veterinary services and pet products was also a challenge, as expressed by some pet owners [5]. Despite the negative implications of Covid-19 on pet and pet-owner relationship, Bowen et al. (2020). revealed that pets contributed significantly to the mental wellbeing of some owners [6]. That is, pet owners were able to cope better with some of the associated emotional adversities of social isolation due to the lockdown restrictions of Covid-19 than non-pet keepers [7,8]. This is so because pet owners had the opportunity to spend more time with their pets [9]. Aside from the social support pets offered during the pandemic [10], the exposure of humans to canine respiratory coronaviruses is postulated to be the driving force behind the immunomodulatory reason why most pet owners experience a mild form of Covid-19 infection [11]. Hence a perfect medical justification for pet keeping during this era, despite the associated economic challenges. However, this is contrary to the position that pets are likely to be marginalised during emergencies e.g., Covid-19 [12].

On March 12, 2020, the first two cases of Covid-19 were reported in Ghana. Consequently, on March 30, 2020, lockdown restrictions were imposed by the government for at least two weeks in some urban areas of the country to limit the reproducibility of the disease based on advice from the Ghana Health Service (GHS). Albeit these lockdown measures, the virus continued to spread unabated. During the partial lockdown restrictions, the already challenged dearth of veterinarians, coupled with the high cost of veterinary services and drugs, further worsened the cost of veterinary care. Subsequently, veterinary services became even more inaccessible [13]. With the rise in the acceptance of pet culture in Ghana, it became imperative to assess the impact of Covid-19, especially the effects of the lockdown and Covid-19 measures, on pet owners and their pets. The researchers are optimistic that this study's findings will provide relevant data to guide policy formulation in preparation for future disasters and for animal welfare and one health advocates to help mount mitigating strategies in unpredictable times.

## 2 Materials and methods

A cross-sectional, descriptive study design approach using structured questionnaires assisted in the investigation of the impact of Covid-19 on pets and pet owners in Accra Ghana. To attain adequate saturation [14], 157 samples were used. Participating hospitals and clinics were in the selected region and provided services to pets and pet owners at least six days a week. For this study, a pet owner was considered to be the individual or group of individuals (owners) that ensure and/or finance the survival and welfare of the pet and provide the basic needs. The questionnaire used is attached as supplementary 1. Participation, both at the veterinary facility and pet owner levels were voluntary and participants were made to sign consent forms prior to answering the questionnaires. A structured questionnaire was administered to generate data for the study. The questionnaire focused broadly on the demographic characteristics of owners; impact of Covid-19 on income, household expenditure and personal difficulties; impact of Covid-19 on the amount spent on the pet, health, and economic impact of Covid-19 on pets and pet owners, and availability of and accessibility to veterinary personnel/professional (veterinarian, veterinary nurse or technician) and pet products. Quantitative data from the study was analysed using IBM Statistical Package for the Social Sciences (SPSS) version 27. Coded data were analysed using descriptive statistics, and the results are presented in the tables below.

## 3 Results

### 3.1 Demographic characteristics of pet owners

Table 1 depicts the demographic features of the pet owners sampled for this study. Most pet owners were males (72.0 %), aged 20–39 (76.4 %). Most pet owners were Christians (84.1 %), and 74 % had higher education. Our findings revealed that most pet owners were employed (56.4 %), with 37.9 % employed and working outside the home.

**Table 1:** Demographic characteristics of the pet owners in this study.

Variable	Category	Percentage
Gender	Male	72.0
	Female	28.0
Age	20–39	76.4
	40–59	21.1
	60 years and above	2.5
Religion	Christian	84.1
	Muslim	8.3
	Traditionalist	1.2
	Others	6.4
Level of education	No formal education	2.5
	Basic education	2.5
	Secondary education	21.0
	Tertiary education	74.0
Occupation	Student	10.8
	Retired	1.9
	Self-employed working outside home	23.6
	Employed working outside home	53.8
	Employed working from home	2.7
	Unemployed	1.9
	Others	5.4

### 3.2 Source of income and expenditure

**Fig. 1:**
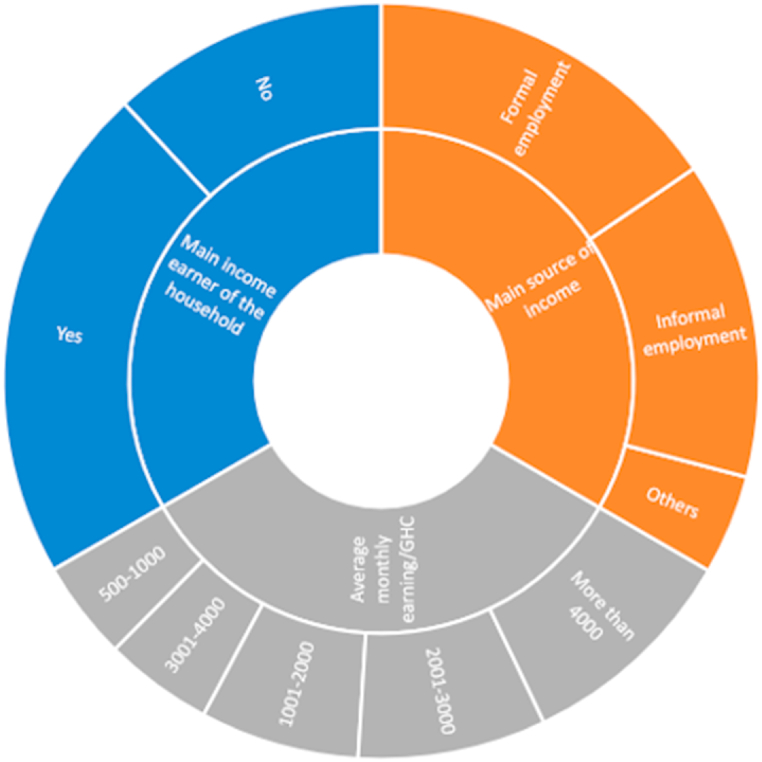
Information on income among respondents.

64.3 % of respondents were the primary earners in their household, while 35.7 % were not. Furthermore, 46.5 % of respondents are in formal employment, 40.8 % in informal employment and 4.5 % are casual employees in registered businesses. Also, 28.7 % of respondents earn more than GHC 4000 per month, while the rest earn less than GHC 4000 per month. However, 82.8 % were not bothered about shortages and did not purchase many dog items before the Covid-19 lockdown phases, whereas 17.2 % were worried about shortages (Fig. 1).

### 3.3 Impact of Covid-19 on income, household expenditure and personal difficulties

**Fig. 2:**
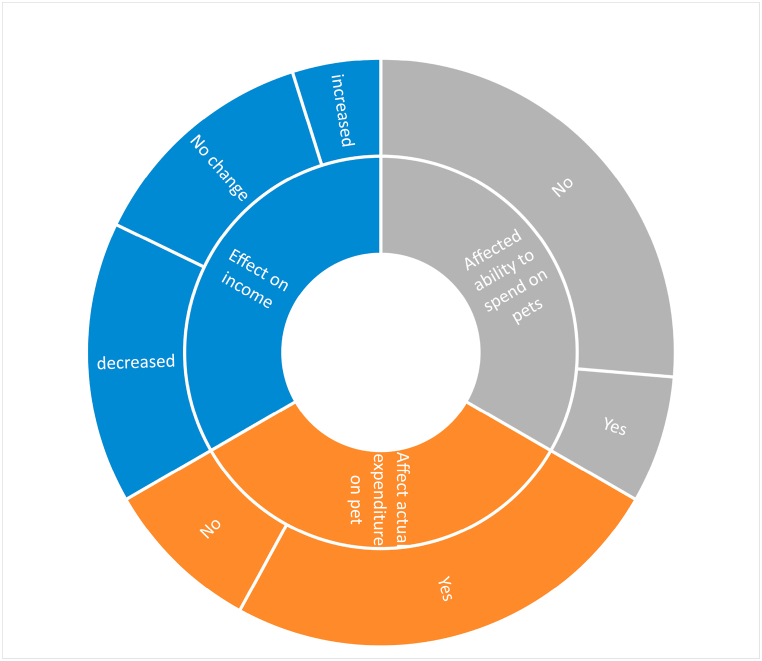
Impact of COVID-19 on pet owners' income, and ability to spend on and actual expenditure on their pets.

46.5 %, had their household income reduced since the outbreak of Covid-19. Furthermore, the influence of Covid-19 on 79.0 % of owners' earnings did not affect their ability to spend on their dogs (Fig. 2). Again, 73.9 % indicated a change in how much they spent on their pets during Covid-19, with 52.2 % increasing their household expenditure during the pandemic. Moreover, 72.5 % showed that the number of essential food items consumed in households had not changed since the outbreak of Covid-19. The results showed that 82.8 % of pet owners experienced no job losses in their households due to Covid-19. For those whose households experienced job loss (17.2 %), the job loss was due to business course (3.8 %), scaling down of business (10.2 %) and non-essential work closure during the pandemic (1.9 %) (Table 2). It was also discovered that Covid-19 improved the living conditions of 68.8 % of pet owners. The main personal obstacles identified by 52.2 % and 25 % of respondents were restricted access to income-earning options and increased security concerns due to the pandemic.

**Table 2:** Impact of Covid-19 on income, household expenditure, and personal difficulties.

Variable	Category	Percentage
Any change in household expenditure during the pandemic	Significantly increased	24.8
	Increased	52.2
	Slightly increased	2.3
	Decreased	3.2
	No change	17.2
Has the quantity of basic food items consumed by the household changed since the outbreak of Covid-19	Increased significantly	11.5
	Increased somewhat	12.7
	No change	72.5
	Decreased somewhat	3.2
Has anyone in your household lost their job during Covid-19	Yes	17.2
	No	82.8
Reasons for losing the job	Laid off because of business closure	3.8
	Laid off due to business scaled down	10.2
	Work considered non-essential during the pandemic	1.9
	Other reason	1.3
Has your household living situation changed due to Covid-19	Improved	68.8
	Worsened	27.4
	No changes	3.8
Describe the main personal challenges you are facing due to the pandemic	(I) Reduced access to income-earning opportunities	52.2
	(II) Increased security concerns	25.5
	Other	7.6
	I and II	14.6

### 3.4 The impact of Covid-19 on various pets kept at home and changes in time spent with pets

**Fig. 3:**
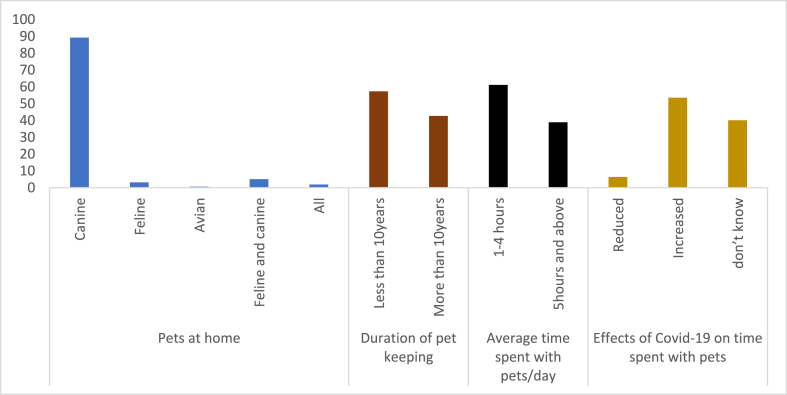
Pets kept at home, duration of pet ownership and the impact of Covid-19 on time spent with pets.

The type of pets kept by pet owners, the duration of pet ownership and the impact of Covid-19 on time spent with pets was interrogated. Most pet owners (89.2 %) who visited the selected veterinary hospitals during the study period kept canine species (Fig. 3). While 57.3 % of pet owners had kept pets for less than 10 years, 42.7 % had owned pets for more than 10 years. 61.1 % of pet owners devoted 1–4 h/day to their pets, while 38.9 % of the respondents spent at least 5 h/day with their pets. Covid-19 increased the time 53.5 % of pet owners spent with their pets.

**Fig. 4:**
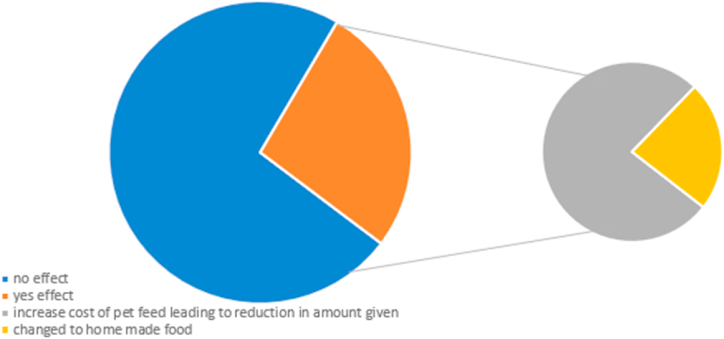
Effect of Covid-19 on pet feed.

Regarding pet feeding, Covid-19 did not affect 73.2 % of respondents, while only 26.8 % were affected. For those (26.8 %) whose ability to feed their pets was affected, (20.4 %) indicated an increase in the cost of feed, which led to a decrease in the quantity given, while 7.6 % indicated a switch from commercial to homemade feed (Fig. 4).

**Fig. 5:**
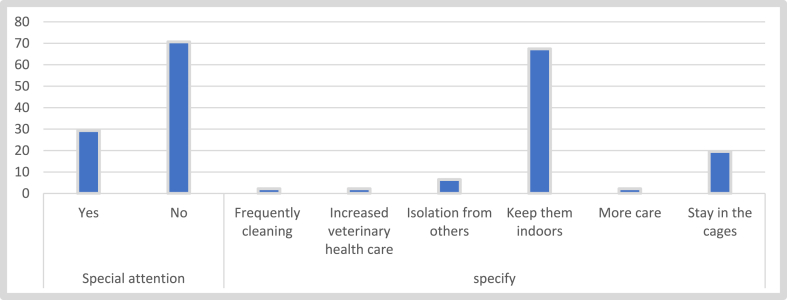
Assessment of pet owners' attention to their pets during the Covid-19 pandemic.

Fig. 5 shows most pet owners (70.7 %) gave no special attention to their pets out of the fear that they might contract Covid-19. However, 29.3 % paid special attention to their pets out of fear of contracting Covid-19. These owners resorted to keeping their pets indoors or cages and preventing them from interacting with other pets as the main modifications in the way their pets were raised prior to the pandemic.

### 3.5 Availability and accessibility of veterinary personnel and measures to mitigate the negative impact of Covid-19

About 60 % of pet owners do not have personal veterinary personnel for their pets (Table 3). Nearly a tenth of those with personal veterinary professionals attending to their dogs at home observed a reduction in the availability of the veterinarian to provide needed services. With respect to the willingness of the respondents to embrace tele-veterinary, 75.2 % were accepting of the concept while 24.8 % were against this notion. Furthermore, 35.0 % of pet owners advocated for the government to provide basic pet care education to prevent diseases and help mitigate the negative impact of future pandemics.

**Table 3:** Availability and accessibility of veterinary personnel and measures to mitigate the negative impact of Covid-19.

Variables	Category	Percentage
Personal veterinary personnel	Yes	41.4
	No	58.6
Has Covid-19 affected their ability to attend to your pets	Yes	9.6
	No	49.0
Willing to embrace tele veterinary	Very unwilling	8.3
	Unwilling	16.5
	Reasonably willing	27.4
	Willing	25.5
	Very willing	22.3
Proposed measures by the government to mitigate the negative impact of future pandemics	(I) Support pet owners with essential medications	19.1
	(II) Providing basic education on pet care to prevent diseases	35.1
	(III) Vet emergency response team across all districts responds urgently to the pet needs at home	5.7
	Others	3.8
	All	7.0
	I and II	25.5
	I and III	1.3
	II and III	2.5

### 3.6 Health and economic impact of Covid-19 on pets and pet owners

Before Covid-19, 57.3 % of pet owners visited the veterinary clinic as and when necessary. Almost all pet owners interviewed (94.3 %) visited the veterinary clinic either as part of their pet's routine visits or for emergency care. The results also demonstrate that 95.5 % of respondents could comfortably afford the cost of veterinary care prior to the outbreak of Covid-19. However, 82.8 % of these respondents could afford the cost of veterinary service during the Covid-19 pandemic, an indication of a 12.7 % reduction. The remaining 17.2 % attributed their inability to afford veterinary products to observed price hikes. Lockdown restrictions and price increments in goods and services affected 14.0 % and 2.5 % of pet owners, respectively. Most pet owners (59.2 %) proposed reducing the price of goods and services to lessen the burden of Covid-19 on the care given their pets. Others suggested education of pet owners on common pet diseases to help animals stay healthy (12.7 %), free pet health check-ups (12.7 %), signing up for pet insurance (5.7 %) and increased veterinary home services (2.5 %) as alternative measures to help relieve the burden on pet owners throughout the Covid-19 pandemic (Table 4).

**Table 4:** Health and Economic Impacts of Covid-19 on pets and pet owners.

Variables	Category	Percentage
Frequency of vet visits prior to Covid-19	Fortnightly	0.6
	Monthly	19.2
	Quarterly	10.2
	Annually	7.0
	When necessary	57.3
	Never	2.5
	Others	3.2
Ability to comfortably afford the cost of veterinary services prior to Covid-19	Yes	95.5
	No	4.5
Ability to comfortably afford the cost of veterinary services during the Covid-19 outbreak	Yes	82.8
	No	17.2
How Covid-19 affected respondents	Increase in prices of goods and services	2.5
	Lockdown restrictions	14.1
	No response	83.4
What can be done to ease pressure on pet owners during the Covid-19 era	Reduces the price of goods and services	59.2
	Education on pet diseases to keep animals healthy	12.7
	Free pet health checks up	12.7
	Consider signing up for pet insurance	5.7
	Increase vet home services	2.5
	Nothing can be done	7.0

## 4 Discussion

### 4.1 Social-demographic characteristics

The ongoing Covid-19 pandemic affects pet owners and likely their companion animals. This study demonstrates how Covid-19 affected the pet owners who participated in the survey and their pets, especially during the lockdown. In this study, most of the pet owners interviewed were males. This finding could be a mere coincidence. Regarding the educational levels and employment status of pet owners in this study, more had tertiary education and were employed. Their educational background and exposure could contribute to their ability to keep and spend on pets. This could also be partly due to the relatively higher incomes associated with tertiary education in Ghana [15].

### 4.2 Impact of Covid-19 on income, household expenditure, and personal difficulties

Congruent with findings from Esam et al. [9] and Pawar et al. [16], this study suggests the Covid-19 pandemic did not affect all pet owners equally. Those whose income increased were most likely considered front liners during the pandemic or whose businesses met increased demand due to the pandemic. Despite the effects on their income, they could still spend money on their pets. Pawar et al. [16] showed that a few pet owners had difficulty covering their dogs' costs. This implied that most pet owners were also not affected economically to the point of neglecting their pets. The possible reason could be attributed to the fact that keeping pets may be considered a luxury by many in Ghana, so people keeping them may be well advised. Regardless, most pet owners will constantly adjust to ensure their pets survive.

The restrictions imposed by the lockdown and its associated effects on the food supply chain led to a modification in purchasing behaviour [17]. As a result, fast food meal sales essentially ceased during stay-at-home orders, but the grocery store and internet seller food sales increased [18,19]. The Covid-19 pandemic did not affect how much basic food was consumed at home [20]. Identical patterns must have occurred in Accra, given that household incomes were not significantly affected. The income levels were most likely kept constant or marginally affected for most houses. Any adverse effect on spending most likely arose from fear of a pending economic crisis rather than a decrease in income. Job losses were attributed to firms scaling down and business closures. Some workers were given leaves with reduced pay as they were regarded as ‘non-essential workers.’ Based on the Pew Research Centre's report by Kochhar and Barroso [21], workers in hotels, retail trade, transportation services, arts, entertainment, and recreation services were at the highest risk of being laid off. While this study did not reveal the category of respondents that lost their jobs, it is likely that those who did belonged to one of these job categories.

### 4.3 Pet kept at home, duration of pet ownership and the impact of Covid-19 on time spent with pets

Most respondents kept dogs (canine species) as pets. Nearly half of the pet owners had been keeping pets for over ten years. The lockdown restrictions provided pet owners with an opportunity to spend more time with their pets-which means a more attachment of pet owners to their pets [9,[22], [23], [24], [25], [26], [27], [28]]. The stay-at-home orders associated with the lockdown restriction in Ghana could be a plausible explanation for why most respondents spent more time with their pets during the Covid-19 lockdown. Even though this study did not focus on the significance of pet-owner interactions during these Covid-19 times, this increase in time spent with pets by owners could potentially be a source of mental and physical support to pet owners in these times of crisis [6,8,26,31]. The increase in time spent reaped benefits for not only owners but the pets as well [22,23,26]. For instance, veterinarians observed that dog owners who spent more time with their dogs provided more care and attention [28]. Thus, we can deduce that since most pet owners spent more time with their pets, they subsequently increased the attention and care they provided.

### 4.4 Impact of Covid-19 on pet feeding

Pet keeping in Ghana is a costly venture. Regardless of this and the negative impact of Covid-19 on pet owners, 73 % of pet owners didn't change how they fed (quantity and quality) their pets. This is in tandem with the findings of Esam et al. [9] and Pawar et al. [16] in New Zealand and India. The study in India even points out that people went out of their way to fend for stray animals. The fear of a shortage of veterinary supplies, including feed, was also reported [16,26].

The increase in feed cost forced nearly a third of all respondents to switch to homemade feed and the regimen. That is, the feeding regimen of pets was greatly influenced by both the financial status of the pet owners and the prevailing spikes in feed prices. The exponential increases in the prices of feed observed during and post-Covid-19 can be attributed to decreases in raw materials for production and kinks in the supply chain of the products. This led to scarcity, driving price hikes [5]. The economic consequences of the pandemic made the affordability of pet feed and other pet needs a concern for pet owners [28].

### 4.5 Assessment of pet owners' attention to their pets during the Covid-19 pandemic

While the predominant mode of Covid-19 transmission is and remains person-to-person (WHO), there is evidence suggesting the possible involvement of companion animals in the transmission of the disease to animals [[29], [30], [31], [32]]. This warrants the need for precautionary measures when dealing with or spending time with companion animals. About a third of the respondents gave special attention to their pets out of the fear that they might contract Covid-19. These individuals kept their pets indoors, considered scheduling more veterinary appointments, isolated their pets from other animals, or increased the grooming and cleaning of their pets and environment. However, special attention given to pets was most likely based on the increased time spent with the pets rather than the fear of contracting the virus. Respondents who provided no extra special care did so under the impression that their pets were not susceptible to Covid-19 or, if so, could not transmit the virus to them.

### 4.6 Health and economic impact of Covid-19 on pets and pet owners

At most, two-thirds of the respondents were the primary earners in their households. In nearly equal parts, formal and informal employment contributed about two-thirds of their income. With an average monthly earning of more than GHC 4000 by nearly a third of the respondents, it is unsurprising that most respondents could afford veterinary services. It further substantiates our previous speculation that perhaps most respondents in this study earned appreciable income to support pet(s) keeping. Regardless of financial uncertainties, including one fostered by the economic crisis due to Covid-19, made pet owners anxious about affording pet food and other pet necessities, accessing and providing medical care if necessary, and keeping their animal's insurance [28].

The difference observed in the patronization of veterinary services was attributed to lockdown constraints, drastic reduction in business sales, and inability to afford veterinary services due to the rapidly increasing prices of products and services. As a result, some pet owners proposed reducing the costs for goods and services and enhancing veterinary home services. Others proposed free health check-ups for pets and the dissemination of information on pet diseases to help keep their animals healthy, while some considered signing up for pet insurance policies to help lessen the financial burden on pet owners during and after the pandemic.

### 4.7 Availability and accessibility of veterinary personnel and measures to mitigate the negative impact of Covid-19

That nearly all respondents can boast of access to veterinary services was encouraging. This contrasts the observation of Applebaum et al. [22] and Kogan et al. [8] that pet owners faced barriers to receiving adequate treatment for their pets due to Covid-19. This is believed to be harmful to the health of animals, especially equine health [33]. Another access route for pet owners to veterinarians and para-veterinarians was tele-veterinary medicine. This involved messaging and voice services to seek veterinary assistance from veterinary professionals. While tele-veterinary medicine is a common practice, Covid-19 brought its advantages to the fore, the least being the reduction of nosocomial diseases since only emergencies are referred to the hospitals. Pet owners were also willing to increase their desire to learn good pet-care tips from the media during the lockdown restrictions. Even with the convenience associated with tele-veterinary medicine, only three fourth of the respondents wanted to take advantage of this opportunity.

As an alternative to telemedicine, a third of the respondent demanded that the government task the relevant authorities with providing basic pet care education to avoid diseases and free pet medication. Less than a tenth of the respondents suggested the institution of veterinary emergency response teams in all districts. These facilities or agents will be available to respond quickly to pet care. Others admonished pet owners to explore the opportunities provided by e-commerce.

### 4.8 Assessment of vitamin supplements

According to Olivindo et al., growing animals fed a meal with a magnesium content below the required levels experienced anorexia, weight loss, hyperextension of the carpal joints, and posterior ataxia 3–6 weeks after feeding [34]. There appears to be a general acceptance of including vitamin supplements in the feed of pets. The impact of Covid-19 did not significantly affect the usage of pet vitamins.

## 5 Conclusion

Covid-19 pandemic generally improved the time spent by most pet owners within the Greater Accra region of Ghana with their pets, with some of these pet owners paying special attention to the general health and well-being of these pets due to fear of them contracting Covid-19. There were also concerns about the increased cost of veterinary service charges and pet feed, although access to veterinary services was readily available. The general increased in prices of products compelled some pet owners to switch from commercial feeds to homemade feeds. The impact of the pandemic on their household income varied depending on the respondent's profession or type of employment. The option of tele veterinary services was proposed as a proactive measure to alleviate the negative impact of future pandemics on pets and pet owners in Ghana.

## Data availability statement

The data from this study has not been deposited into any public repository and will be made available upon request.

## CRediT authorship contribution statement

**Godwin Dogbey:** Methodology, Investigation, Data curation, Conceptualization. **Amos Dugah:** Writing – original draft, Data curation. **Richard Kwamena Abbiw:** Writing – review & editing, Writing – original draft, Visualization, Methodology, Formal analysis, Conceptualization. **Anthony Agbolosu:** Writing – review & editing. **Kweku Asare-Dompreh:** Validation, Formal analysis. **Theophilus Odoom:** Writing – review & editing, Conceptualization. **Allen Okine:** Writing – review & editing, Methodology. **Jonathan Amakye-Anim:** Writing – review & editing, Supervision. **Hope Richard Otsyina:** Writing – review & editing, Resources. **Ben Enyetornye:** Visualization, Methodology, Formal analysis, Conceptualization.

## Declaration of competing interest

The authors declare that they have no known competing financial interests or personal relationships that could have appeared to influence the work reported in this paper.
