# Supplementary material for: Impact of COVID-19 on pets and pet owners: A survey conducted in selected veterinary clinics in Accra, Ghana
Source: Heliyon. 2024 Sep 4;10(17):e37328. doi: 10.1016/j.heliyon.2024.e37328 (PMC11409105; doi:10.1016/j.heliyon.2024.e37328)
Supplement: Multimedia component 1 [file mmc1.docx]

**QUESTIONNAIRE ON IMPACT OF COVID-19 ON PETS AND PET OWNERS IN ACCRA, GHANA.**

**Introduction and seeking of respondent’s consent.**

Dear Sir/Madam, I would like to thank you for agreeing to respond to this questionnaire. I am Amos Dugah a veterinary nursing student at the University for Development Studies. I am conducting a study to determine the impact of COVID 19 on pets and pet owners in Accra.

The information you will provide in this questionnaire will be used for research purposes only. I would also like to assure you that the information provided in response to these questions will be handled with strict confidentiality. Please note that your participation in this research is voluntary and refusal to participate will involve no penalty or loss of benefits to which you are otherwise entitled. Should you have more questions about this research, kindly contact the Principal Investigator of this project whose contact is provided below.

Do you consent to take part in this research study? 1. Yes

2. No

Principal Investigator: Dr Godwin Dogbe

Telephone: +233579177386\+233542206468

E-mail: gdogbe@uds.edu.gh

Date: ……………………………..

**DEMOGRAPHICS**

Contact of respondent………………………………

1. Area of residence …………………………
2. Age of respondent
   1. 20 – 39 years
   2. 40 – 59 years
   3. Sixty years and above
3. Gender of respondent
   1. Female………
   2. Male …………….
4. Level of education
   1. No formal education
   2. Basic education
   3. Secondary/Technical high school
   4. Tertiary education
5. Religion
   1. Christianity
   2. Islam
   3. Traditional
   4. Others (specify) …………………
6. Employment
   1. Student
   2. retired
   3. Self-employed working outside of home
   4. Self-employed working from home
   5. Employed working outside of home
   6. Employed working from home
   7. Furloughed due to Covid-19
   8. Redundant due to Covid-19
   9. Unemployed
   10. Others (specify)…………………………………………

**PET INFORMATION**

1. How many pets do you have at home?
2. What species of pets do you keep?
3. Based on your response to Q8 above, what breeds of pets do you have?
4. How long have you been keeping pets?
5. On average, how much time do you spend with your pets in a day?

**INFORMATION ON PETS DURING COVID-19**

1. Has covid 19 affected the time you spend with your pets in any way? Yes/No
2. If yes to Q12 above, how did Covid-19 affect the time spent with your pets
   1. I have reduced drastically the time I spend with my pets due to fear of contracting Covid from them
   2. I spend more time with my pets because I was always home due to the lockdown
   3. others
3. Has covid 19 in any way affected the diet/feeding of your pets? Yes/No
4. If yes to Q14 above, how?....................................................................................................
5. Are you currently giving any special attention to your pets due to fear of them contracting Covid-19? Yes/No
6. If yes to Q16 above, please specify?...................................................
7. Do you see owning a pet during the Covid-19 era as burdensome? Yes/No
8. Have you considered purchasing a new pet during this Covid-19 era? Yes/No

**PETS, COVID 19 AND VETERINARY SERVICES**

1. How often do you visit or call the vet to check your pets before covid19?
2. Has covid 19 affected the number of visits in any way? Yes/No
3. If yes to question 20 above, please specify…………………………..
4. Are you able to comfortably afford the cost of veterinary services before the outbreak of Covid-19? Yes/No
5. Are you able to comfortably afford the cost of veterinary services after the outbreak of Covid-19? Yes/No
6. If no to question 23 above, what accounted for your inability to pay for such services?
7. What do you think can be done to help ease the pressure on pet owners in terms of the cost of veterinary services during this covid-19 era?
8. Do you have a personal veterinary doctor? Yes/No
9. Has Covid-19 affected his/her availability to attend to your pets? Yes/No
10. Are you willing to embrace tele-veterinary medicine in pandemic situations like this?
11. Very unwilling
12. Unwilling
13. Reasonably willing
14. Willing
15. Very willing
16. What policies do you think the government should put in place to help reduce the negative impact of future pandemics on pets?
17. Should support pet owners with basic medications (e.g., dewormers and vitamin supplements) and feed
18. Provide basic education on pet care to prevent diseases
19. Veterinary emergency response team across all districts to urgently respond to pet needs at home
20. Others…………….
21. During the COVID-19 pandemic, how has your interest in learning healthy pet-keeping tips from the media (newspaper articles/magazines blogs/videos/TV shows/text messages) changed?
22. Significantly increased
23. Slightly increased
24. Grossly similar
25. Slightly decreased
26. Significantly decreased
27. Do you normally provide vitamin supplements to boost your pet’s immune system? Yes/No
28. If yes to question 32 above, how has providing supplements to boost your pet’s immunity changed?
29. Significantly increased
30. Slightly increased
31. Grossly similar
32. Slightly decreased
33. Significantly decreased
34. Before the lockdown phase of COVID-19, did you buy lots of dog products with the fear that there may be a shortage? Yes/ No

INCOME

1. Are you the main income earner of the household? (Yes/No)
2. What is your main source of income?
3. Formal employment (salary/wage)
4. Informal employment
5. Casual employee in a registered business
6. Others, ……………………………………
7. On average, how much do you earn monthly?
8. GHC 500-1000
9. GHC 1001 -2000
10. GHC 2001-3000
11. GHC3001-4000
12. GHC 4001 or more
13. On average, how much were you spending per month on household expenditures such as food, medical, utilities before the pandemic?
14. GHC
15. GHC
16. GHC
17. GHC 5000 or more
18. Has there been any change in household expenditure during the pandemic?
19. Significantly increased
20. Increased
21. significantly decreased
22. Decreased
23. No change
24. How much on average do you spend on pet products (feed, shampoo, dewormers, chain/leash, cage etc.) per month?
25. Since the outbreak of Covid-19, how has your income been affected?
26. Increased
27. Decreased
28. No change
29. Has there been any change in how much you spend on your pet(s) during the pandemic? (Yes/ No)
30. If yes, by how much?
31. Has the effect on your income impacted your ability to spend on your pet(s)? (Yes/No)
32. Yes, it has had an impact.
33. No, it hasn't had an impact.
34. How many other people are gainfully employed in your household?............
35. Has the quantity of basic food items consumed by the household changed since the outbreak of covid-19?
36. Increased significantly
37. Increased somewhat
38. No change
39. Decreased somewhat
40. Decreased significantly
41. Has anyone in your household lost their job due to Covid-19? (Yes/No)
42. If you answered yes to the previous question, what was the reason for losing their job?
43. Laid off because business closed
44. Laid off because business scaled down
45. His/her work considered as non-essential during the pandemic
46. Others
47. Are you confident that those who have lost their jobs in your household will be able to get a job/return to their former jobs when the pandemic is over?
48. Yes
49. Not sure
50. No
51. Other
52. Has your household's living situation changed due to Covid-19? (Improved/Worsened)
53. Describe the main personal challenges you are facing due to the pandemic.
54. Reduced access to income earning opportunities
55. Increased security concerns
56. Child abuse (within your household)
57. Abuse (physical/verbal/emotional)
58. Others………………………………….
